# Reducing Oil Separation in Ready-to-Use Therapeutic Food

**DOI:** 10.3390/foods9060706

**Published:** 2020-06-01

**Authors:** Andrea Zuzarte, Melody Mui, Maria Isabel Ordiz, Jacklyn Weber, Kelsey Ryan, Mark J. Manary

**Affiliations:** Department of Pediatrics, Washington University, St. Louis, MO 63110, USA; andrea.zuzarte@wustl.edu (A.Z.); mkmmui@gmail.com (M.M.); miordiz@wustl.edu (M.I.O.); jacklyn.weber.10@gmail.com (J.W.); kelseynryan@gmail.com (K.R.)

**Keywords:** ready-to-use therapeutic food, oil separation, hydrogenated vegetable oils, food aid, peanut paste

## Abstract

Ready-to-use therapeutic food (RUTF) is a shelf-stable, low moisture, energy dense medicinal food composed of peanut butter, vegetable oils, milk powder, a multiple micronutrient premix and sugar. RUTF is used by millions of children annually to treat malnutrition. After mixing, RUTF is a semisolid covered with oil. To produce a homogenous RUTF, hydrogenated vegetable oils are incorporated in small quantities. This study utilized a benchtop methodology to test the effect of RUTF ingredients on oil separation. An acceptable oil separation was <4%. This method compared 15 different vegetable oil stabilizers with respect to oil separation. The dynamic progression of oil separation followed a Michaelis–Menten pattern, reaching a maximum after 60 days when stored at 30 °C. Hydrogenated vegetable oils with triglyceride or 50% monoglycerides reduced the oil separation to acceptable levels. The additive showing the largest reduction in oil separation was used in an industrial trial, where it also performed acceptably. In conclusion, fully hydrogenated soybean and rapeseed oil added as 1.5% controlled oil separation in RUTF.

## 1. Introduction

Ready-to-use therapeutic food (RUTF) is recommended as the primary food for children with severe acute malnutrition [1]. RUTF is a mixture of peanut paste, vegetable oil, sugar and milk powder fortified with vitamins and minerals. RUTF is a lipid-based emulsion which contains cooked, dry ingredients suspended in an oil matrix. The water activity of RUTF is extremely low, 0.2 to 0.5, making it refractory to most microbial contamination [2,3]. This food formulation has made RUTF extremely versatile, in that it can be safely used in many emergency and adverse circumstances. Foods with similar formulations are commonly used as supplemental products among vulnerable populations, such as moderately malnourished children [4,5], wasted individuals with human immunodeficiency virus infection [6,7,8,9] and malnourished pregnant women [10,11]. Ready-to-use products are produced in places that are remote from the people that consume them. RUTF is designated as a medicinal food, purchased and distributed by UNICEF, government health services and not-for-profit charities. RUTF must be of a soft, semi-solid consistency so that it can safely and easily be consumed by infants. Many stabilizers increase the viscosity of RUTF [12].

UNICEF sets the product standards for RUTF, which include that there will be no visible oil separation and a shelf life of 2 yrs [2]. Oil separation refers to the natural tendency for oil and solids in RUTF to partition over time, creating a viscous semi-solid covered by a layer of oily liquid. This oil separation standard is difficult to achieve, and to that end, UNICEF allows for the addition of stabilizers in quantities of up to 2% of the RUTF. The stabilizers are restricted to those approved for use in infant foods according to the CODEX Alimentarius. Approved stabilizers include lecithins, palm oil, triglycerides, diglycerides and monoglycerides. Lecithins are found in animal and plant membranes, and their amphiphilic properties allow them to hold together hydrophilic and hydrophobic aggregates in the food matrix. Glycerides are created by the hydrogenation of vegetable oils, and are usually sold as proprietary products of a non-divulged composition. The ambient conditions for RUTF storage and use are typically 30 °C, as much of the food is used in sub-Saharan Africa and south Asia. UNICEF encourages the testing of oil separation in RUTF at 40 °C, which they assert is a condition that accelerates the oil separation, and provides information as to whether the RUTF will meet their shelf-life specification. 

This investigation will systematically assess the addition of a variety of commercial stabilizers in RUTF to better understand how oil separation might be controlled.

## 2. Materials and Methods 

The experimental strategy was to make RUTF using a reproducible, standardized benchtop procedure, and to incorporate varying amounts of stabilizer to observe the effect on oil separation, taking care to use only stabilizers that meet UNICEF specifications in terms of content and amount. The tiered nature of the strategy was to first screen many stabilizing agents for their effectiveness over a two-week period on the benchtop, followed by a more prolonged investigation of the stabilizers with the least oil separation. Two different storage temperatures were chosen for the RUTF for a 6-month period. Finally, the best performing emulsifier was chosen for an industrial trial.

### 2.1. RUTF Ingredients

Non-fat dry milk (NFDM) Grade A was purchased from Jacoby and Company, Inc. (St. Louis, MO, USA); peanut butter without stabilizers was donated by Birdsong Peanuts/Olam Edible Nuts (Edenton, NC, Canada); sugar was purchased from Domino Foods Inc. (Nashville, TN, USA); palm olein oil was purchased from Ghana Palm Oil Development Company (Kwae, Eastern Region, Ghana); canola oil was purchased from Soaper’s Choice (Des Plaines, IL, USA); vitamin and mineral premix specially formulated for Project Peanut Butter was purchased from DSM (Schenectady, NY, USA); stabilizers were donated by Lonza Group (South Plainfield, NJ, USA), Corbion N.V. (Lenexa, KS, USA) and DuPont Danisco (New Century, KS, USA). 

The vitamin and mineral premix was configured by Project Peanut Butter (PPB) to meet the UNICEF specifications. It was produced at DSM (Isando, South Africa). It included calcium, iron, magnesium, phosphorus and zinc in mg quantities and copper, selenium, vitamin A, vitamin E, vitamin D3, vitamin K, niacinamide, pantothenic acid, vitamin B1, vitamin B2, vitamin B6, vitamin B12, vitamin C, folic acid biotin and iodide in µg quantities. The mixture is not proprietary and is available upon request from the corresponding author. Given the polar nature of most of these micronutrients, and the fact that they were being mixed into a lipid/solid matrix, it was assumed that they were not contributing to the visible oil separation.

### 2.2. Benchtop RUTF Preparation and Storage

The standard RUTF formulation was produced on the benchtop in order to best replicate the processing variables and conditions of African RUTF production facilities [13]. The RUTF was composed of 27% peanut paste, 25% non-fat dry milk powder, 15.48% palm oil, 2.9% soybean oil, 26.7% sugar and 2.92% vitamin and mineral premix. To this formulation, varying amounts of stabilizers were added. Heat was applied to the vegetable oils and the additive via a hot plate until the stabilizer was dissolved. All subsequent processing occurred in a double-walled heated bowl with slow, continuous agitation via a paddle mixer. Peanut paste was initially mixed to 52.5 °C (±2.5 °C) to ensure homogeneity, after which the dissolved additive in the palm and soy oils was added and mixed for 10–15 min at 50 °C (±5 °C). The remaining dry ingredients, sugar, dairy powders and micronutrient premix, were added gradually to the liquid mixture, and its temperature was monitored until it reached 50 °C (±5 °C). The fully incorporated RUTF was then mixed for an additional 30 min at 50 °C (±5 °C). Packaging immediately followed the RUTF production. Quantities of 200 g (±0.5 g) RUTF were packaged in 250 mL wide mouth bottles, transferred carefully to ensure the RUTF was compact with no air bubbles or disruptions which could affect the amount or interpretation of oil separation. Storage conditions allowed for temperature control and added moisture to mimic the warm and humid long-term storage conditions of African RUTF facilities. 

No nutrient compositional testing was done on these test mixtures of RUTF, as they were identical for all experiments. This was the same recipe used at PPB at the time these investigations were begun in 2016. This recipe yielded a RUTF with 548 kcal/100 g, with 15.75% protein, 31.6% lipid, 7.2% omega 6 polyunsaturated fatty acid and 0.3% omega 3 polyunsaturated fatty acid.

UNICEF recommends an accelerated stability study, conducted at 40 °C, to indicate what oil separation may be seen in RUTF stored for >1 yr. Thus, we stored RUTF at both 30 °C and 40 °C in this investigation. 

### 2.3. Vegetable Oil Stabilizers to Reduce Oil Separation

Fifteen commercial vegetable oil-based stabilizers were tested with respect to their ability to reduce oil separation after one week and storage at 30 °C (Table 1). The information in Table 1 was obtained from the product data sheets from the additives. These included lecithins, palm oils, triglycerides, diglycerides, 50% monoglycerides and 90% monoglycerides. For the purposes of a concise description, these vegetable oil stabilizers were assigned a letter A through O. The amount of additive in the RUTF used in this initial screening trial was 1.5% of commercial product in the mixed RUTF. The four stabilizers that showed no oil separation in the 7-day screening trial were then tested at three different concentrations, 0.5%, 1.0% and 1.5%, and a storage for 6 mos at 30 °C and 40 °C. 

To better understand the kinetics of the oil separation, RUTF with 1.0% of the four best performing stabilizers was compared over time. The concentration of 1.0% was chosen to allow for the clear observation of different amounts of oil separation at different time points. This allowed for an accurate determination of the shape of the oil separation curves. The resultant curves were then modeled to identify the best fit mathematical relationship with respect to time. 

### 2.4. Performance at Industrial Scale

On the basis of the bench top effectiveness, a single stabilizer was tested in several mixes in industrial scale machinery. The industrial process has three phases, described below.

1. All dry ingredients were added into a large ribbon blender in the same sequence as was done in the benchtop method. No external heat was added to the mixture other than the preheating of the vegetable oil and stabilizers to 65 °C. The RUTF was mixed for 25 min.
2. The RUTF was transferred from the ribbon blender to a 50 °C water-jacketed, constantly stirred holding tank. In the transfer process, the RUTF passes through a vertical, conical grinder with a 3 cm orifice and high-speed rotating bit. The purpose of the grinder is to disperse aggregates of milk powder or other dry matter into the lipid liquid.
3. The passage of the heated, stirred mixture through a disk mill and then into the packaging machine. The temperature as the RUTF passed out of the disk mill was 70 °C.

This work was conducted at the Project Peanut Butter Malawi facility. This was done to determine if the increased temperature and energy from grinding altered the observed oil separation, as it said that such conditions are needed to activate some of the hydrogenated vegetable oil stabilizers. 

Oil separation was measured after 1, 14, 28, 42, 56, 84 and 112 d after packing and storage at 30 °C and 40 °C.

### 2.5. Definition and Measurement of Oil Separation in RUTF 

The amount of oil separation in RUTF was quantified by measuring the height of visible oil over the total height of the sample in the 250 mL bottle used to store the product. This was deemed an effective method as the oil was the least dense ingredient and would separate per gravitation in an even manner in the selected packaging. The total height of product in the cylindrical container and the thickness of the oil layer were measured with a metal ruler to the nearest mm. A percentage of the oil separation was calculated for each sample. The oil separation was measured at designated time points after mixing and storage. The differences in oil separation for identical replicates were tabulated to calculate the SD of the measurement. When comparing the oil separation in RUTFs composed with different stabilizers, only differences > 3 SD were considered to be significant. 

To quantify the visual standard prescribed by UNICEF, preparations of RUTF with 0% to 6% oil separation were made and visually examined by the investigators. Using two criteria, (1) that the product upheld a soft–semisolid consistency and (2) that obvious oil pooling was not present, an arbitrary level of oil separation that corresponded to <4% oil separation using the height measurement method was chosen as an acceptable amount of oil separation.

## 3. Results 

### 3.1. Variability of Experimental Method and Dynamic Nature of Oil Separation

Of the 144 different RUTF formulations for which replicate samples were prepared, the SD varied from 0% to 0.35%, demonstrating the reproducibility of the benchtop method. Therefore, when comparing oil separation between different formulations, only differences >1% were considered to be significant.

### 3.2. Effect of Vegetable Oil Stabilizers in RUTF to Reduce Oil Separation

**Figure 1:**
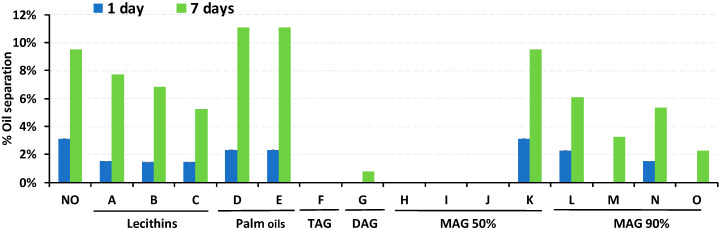
Oil separation in RUTF containing each of the 15 different stabilizers tested during 7 days at 30 °C. The results are displayed by category of the additive and letter code, and the abbreviations used are triglyceride (TAG), diglyceride (DAG) and monoglyceride (MAG). Letter codes are described in Table 1.

Oil separation in RUTF made with 1.5% of one of fifteen hydrogenated vegetable oils was assessed over 7 days at 30 °C (Figure 1). Four stabilizers showed no separation; F, H, I and J. Three of these stabilizers contained 40%–60% monoglycerides, and the fourth contained mostly triglycerides. Palm oils did not reduce oil separation, lecithins reduced oil separation by 19%–45% and stabilizers with 90% monoglycerides reduced oil separation by 36%–76%. These categories of stabilizers were considered inferior, and no further testing was undertaken with them. These benchtop observations are consonant with the industrial experience of Project Peanut Butter.

**Figure 2:**
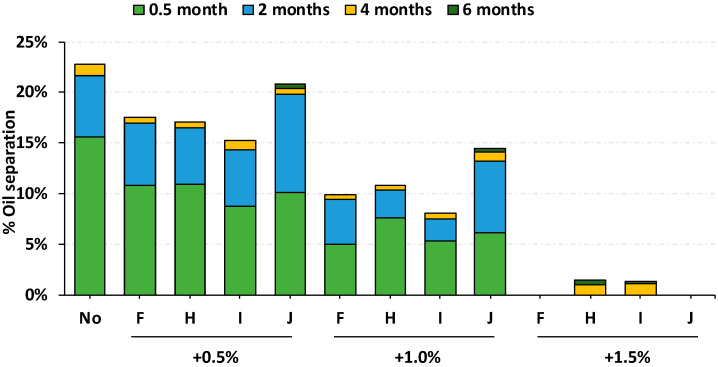
Comparison of oil separation at 30 °C over a 6-month period for the four most effective stabilizers selected in Figure 2. Each stabilizer was tested for three different composition amounts, 0.5%, 1.0% and 1.5%. Abbreviations are triglyceride (F) and 50% monoglyceride (H, I, J). The stabilizers are described in more detail in Table 1.

A 6 mos-oil separation study was undertaken using three different amounts of F, H, I and J; 0.5%, 1.0% and 1.5% of the RUTF composition. All of these hydrogenated vegetable oils markedly reduced oil separation in RUTF for 6 mos when incorporated as 1.5% of the RUTF (Figure 2). The use of any of these stabilizers in amounts <1.5% achieved only modest reductions in oil separation: a < 50% reduction.

**Figure 3:**
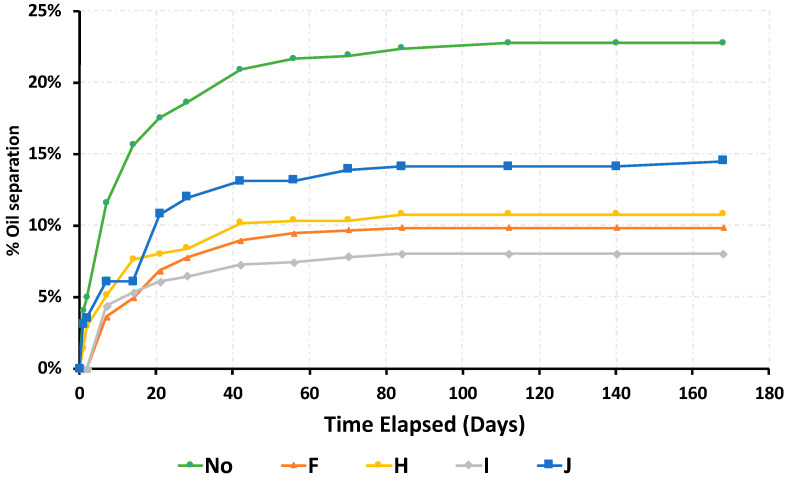
Dynamic pattern of oil separation seen in RUTF containing no stabilizers (No) compared to Table 1. The addition of four hydrogenated vegetable oil stabilizers (F, H, I, J) during six months of storage at 30 °C. Letter codes are described in Table 1.

The dynamic pattern of oil separation is shown in Figure 3. Differences in the oil separation in RUTFs containing no additive or 1% of different stabilizers, at 30 °C storage, were evident after 20 days, and the oil separation approached an asymptotic limit by 60 days.

The oil separation followed a Michaelis–Menten pattern described by the following equation:

(1) $$\mathrm{Oil}\;\mathrm{separation}=\frac{\left(\mathrm{maximum}\;\mathrm{separation}\right)*\left(\mathrm{time}\right)}{\left(\frac{\mathrm{maximum}}{2}\right)+\left(\mathrm{time}\right)}$$

### 3.3. Comparison of Oil Separation in RUTF at 30 °C and 40 °C

**Figure 4:**
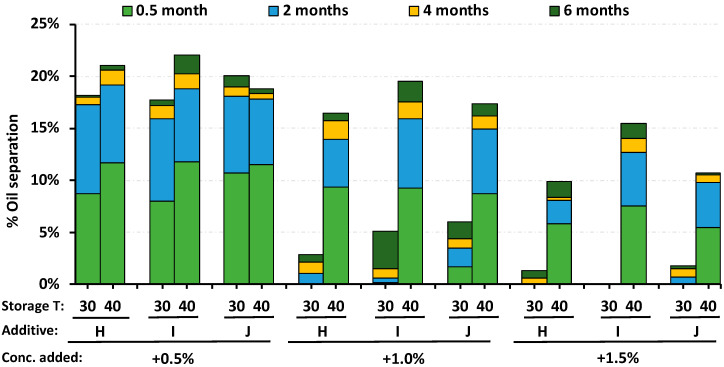
Comparison of oil separation in RUTF at 30 °C and 40 °C for nine RUTF preparations made with three different 50% monoglyceride stabilizers (H, I, J) used at three different concentrations (0.5%, 1.0% and 1.5%). The stabilizers are described in Table 1.

To determine the relationship between oil separation under the accelerated conditions and a typical storage at 30 °C, three RUTF formulations were compared at 30 °C and 40 °C for 6 mos. The three different RUTFs were formulated using the three best 50% monoglyceride stabilizers, H, I and J; the findings are shown in Figure 4. Among RUTF mixtures with little appreciable reduction in oil separation (0.5% of stabilizer), the differences seen between 30 °C and 40 °C were nominal. For oil separation values between 3–6% (1% of stabilizer), storage at 40 °C was 4.2-fold greater than at 30 °C. For a nearly complete reduction of the oil separation (1.5% of stabilizer), the oil separation at 40 °C was 6.7-fold greater than at 30 °C. 

### 3.4. Industrial Scale Performance of Best Performing Additive to Reduce Oil Separation

**Figure 5:**
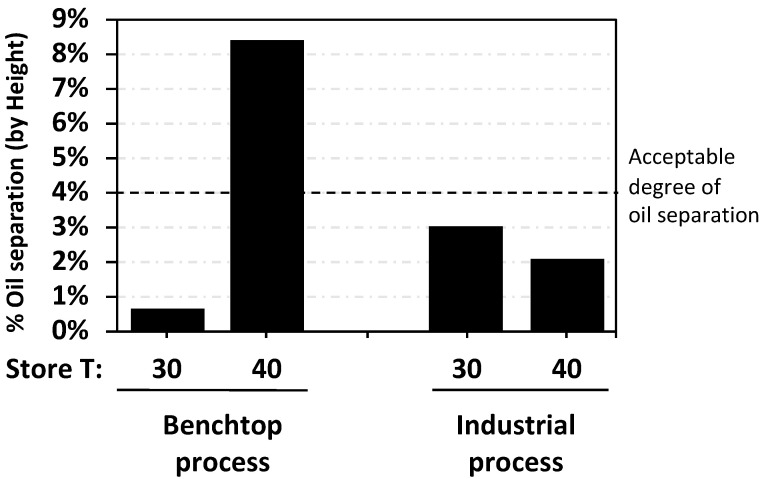
Comparison of oil separation of RUTF mixed with 1.5% of stabilizer H using the benchtop method and the industrial process and stored for 4 mos at 30 °C or 40 °C.

Stabilizer H, one of the 50% monoglyceride hydrogenated vegetable oils, was chosen for an industrial trial. Two hundred kilograms of standard RUTF recipe with 1.5% of H was processed through the Malawi Project Peanut Butter system, and the samples were collected, stored at 30 °C and 40 °C for 4 mos and assessed for oil separation (Figure 5).

## 4. Discussion

This study demonstrates that commercial lipid-based stabilizers composed of either triglycerides or 50% monoglycerides achieve a superior reduction in oil separation in RUTF stored at 30 °C when compared to other common commercial stabilizing agents. The kinetics of oil separation follows a Michaelis–Menten pattern with a maximum separation achieved at 60 d, and thus oil separation can be adequately assessed with a 2-mos study under actual storage conditions. Oil separation at a storage temperature of 40 °C does not follow a similar pattern as that at 30 °C.

The data from the industrial trial emphasize a major limitation of our study. The work was largely a benchtop exercise, and only one additive was tested with vigorous industrial milling. Milling reduces the particle sizes of sugar and increases the temperature of the mixture briefly as it passes through the disks. We have no direct information as to how the other stabilizers would perform under large-scale conditions. The industrial process seems to reduce the discrepancies seen between benchtop RUTFs stored at 30 °C and 40 °C. The benchtop exercise does replicate the gentle mixing achieved by ribbon blenders which are routinely used to achieve most of the mixing in RUTF production.

The kinetic observations indicate that oil separation in RUTF is a process of molecular migration of fats resulting in the aggregation of fats of similar molecular configuration.

It is interesting to note that the 50% monoglyceride stabilizer made from oleate, a monounsaturated fatty acid, did not prevent oil separation (K in Figure 2), while a similar stabilizer made from stearate, a saturated fatty acid, reduced oil separation very well. 

One stabilizer, F, was characterized as a triglyceride (Figure 2). F is industrially produced in a similar manner as the mono- and diglycerides stabilizers, and the physiochemical mechanism of reduction in oil separation in stabilizer F is similar to the distilled monoglycerides. One regulatory difference is that triglycerides are categorized as nutrients, not additives, by CODEX Alimentarius, so the amount of F in RUTF is not limited to 2%.

One rationale offered for an accelerated stability study is that the results at 40 °C reflect what would happen at 30 °C over a longer period of time. It is asserted by UNICEF that oil separation increases linearly with storage time, and the accelerated stability study is reflective of RUTF stability for a time period that is twice the duration of stability at ambient conditions. In practical terms this means the stability found for 6 mos at 40 °C would be similar to that at temperatures of 25–30 °C for 12 mos. Our data are not consistent with the notion that oil separation increases linearly with time, but rather that it plateaus after 60 d. The findings from the study suggest that the physiochemical processes governing oil separation are different at 40 °C and not predictive of what might be seen if RUTF were stored at 30 °C for a longer period of time. The melting point of the primary fat in peanut butter is 43 °C, which is close to 40 °C, and this may explain why oil separation at 30 °C and 40 °C is disparate. The typical storage conditions for programs distributing RUTF in Africa and south Asia are 25–32 °C, but rarely 40 °C, which also puts into question the relevance of an accelerated stability study. The benchtop method storage temperature at 40 °C resulted in a nine-fold increase in oil separation, while for the industrial process, oil separation was similar at 30 °C and 40 °C. The observed oil separation in RUTF made using the benchtop process stored at 30 °C agreed with the industrial process; both found a separation of <3%. Our observations do not identify a utility for oil separation storage studies at 40 °C. 

Peanut paste in RUTF is not prescribed on a regulatory basis by UNICEF. There have been recipe formulations and even clinical trials with RUTF made without peanut [3,14,15]. Oil separation may well be very different in these RUTF formulations, as peanut is composed of 50% oil, and the majority of this oil is omega6 polyunsaturated fatty acids. However, these ‘peanut free’ RUTF formulations have never gone on to be put into operation, due to inferior clinical outcomes. Thus, at present, all RUTF contains peanut, and oil separation in this context must be addressed.

Recently, a RUTF without a hydrogenated vegetable oil stabilizer was used in a large clinical trial [16]. This RUTF contained oat, as well as a reduced peanut and sugar content. The results demonstrated a clinical superiority for this stabilizer-free oat-RUTF; oat-RUTF increased recovery and decreased death. Oil separation was controlled by the β-glycan carbohydrate in oat, which prevented the oil from pooling on the top of the solids and held it within the food matrix. This innovation has yet to be put into operation but may reduce the need for hydrogenated vegetable oil additives in RUTF.

The focus of this investigation was on the performance of hydrogenated vegetable oil stabilizers on oil separation in RUTF. Oil separation is a standard set by UNICEF, the primary purchaser of the product. It is important to keep in mind that the consumer of RUTF is a severely malnourished child. Other important considerations are the effects of these stabilizers on RUTF consumption and the gut health of the vulnerable child [17,18,19]. Future investigations of oil separation in RUTF should consider not only the specifications of the intermediate purchaser of RUTF, but also the consumer.

## 5. Conclusions

1. The dynamic nature of oil separation in RUTF follows a Michaelis–Menton pattern with a maximum separation seen after 60 d.
2. Triglyceride and 50% monoglyceride stabilizers made from hydrogenated vegetable oils achieved an oil separation <4% in RUTF on the benchtop and on an industrial scale.
3. When RUTF is stored at 40 °C, instead of the typical storage at 30 °C, the oil separation is greater and follows a pattern that differs from the industrial RUTF that is in widescale use. We find no reason to recommend stability testing at 40 °C for the purposes of assessing oil separation.

## Figures and Tables

**Table 1:** Stabilizers tested to reduce oil separation in ready-to-use therapeutic food.

Additive Type	Mono-Glyceride Content	Free Fatty Acid (max)	Free Glycerin (max)	Iodine Value	Ingredient Form	Melting/Dropping Point	Trans Fat	Additive Product Name
***Lecithins***								
**A.** Soy Lecithin	Not stated	Not stated	Not stated	Not stated	Liquid	N/A	Not stated	Generic Soy lecithin
**B.** Deoiled soy lecithin	Not stated	Not stated	Not stated	Not stated	Powder	N/A	Not stated	Lecigran™ 1000 P
**C.** Soy lecithin	Not stated	Not stated	Not stated	Not stated	Powder	N/A	Not stated	Metarin^®®^ P
***Palm oils***								
**D.** Refined, bleached and dried palm oil	<3%	<0.1%	Not stated	58	Liquid-solid	22 °C	Not stated	Generic Palm Olein
**E.** Non-hydrogenated, modified, liquid palm oil	<3%	0.05%	Not stated	Not stated	Liquid	20 °C	<1%	Durkex™NT 100-MB
***Triglycerides***								
**F.** Fully hydrogenated rapeseed oil	3%–8%	0.50%	Not stated	3 g I2/100 g	Pellets	61 °C	Not stated	Palsgaard^®®^ 6111 pellets
***Diglycerides***								
**G.** Soy lecithin	3%–8%	1%	1.50%	1.5	Bead	70 °C	<0.1%	Trancendim^®®^ 180
***Monoglyceride composition about 50%***								
**H.** Fully hydrogenated soybean oil	≥42%	1%	1%	3	Beads	60–65 °C	Not stated	BFP^®®^ 74K
**I.** Refined fully hydrogenated vegetable fat blend	40%–60%	1.5%	2%	2	Beads	64 °C	<0.5%	Grindsted^®®^ Mono-Di HV 52 K-A
**J.** Hydrogenated soybean oil	≥52%	Not stated	2%	Not stated	Beads	57–62 °C	<0.1%	Aldo™ HMS KFG
**K.** Hydrogenated vegetable oil	46%	Not stated	1.5%	Not stated	Liquid-solid	19–23 °C	Not Detected	Aldo™ MO KFG
***Monoglyceride composition about 90%***								
**L.** Fully hydrogenated soybean oil	90%	1.50%	1%	1	Beads	72 °C	< 0.5%	Dimodan^®®^ HS K-A
**M.** Fully hydrogenated palm-based oil	90%	1.50%	1%	1	Beads	69 °C	<1%	Dimodan^®®^ HP US MB
**N.** Hydrogenated soybean oil	90%	1.50%	1.20%	1.2	Bead	72 °C	Not stated	Alphadim^®®^ 90 SBK
**O.** Rapeseed	90%	Not stated	1%	1	Powder	80 °C	<1%	Grindsted^®®^ Crystallizer 110 R Kosher
